# Ultrahigh resolution radiation imaging system using an optical fiber structure scintillator plate

**DOI:** 10.1038/s41598-018-21500-z

**Published:** 2018-02-16

**Authors:** Seiichi Yamamoto, Kei Kamada, Akira Yoshikawa

**Affiliations:** 1 Radiological and Medical Laboratory Sciences, Nagoya University Graduate School of Medicine, Nagoya, Japan; 2 New Industry Creation Hatchery Center (NICHe), Tohoku University, Sendai, Japan

## Abstract

High resolution imaging of radiation is required for such radioisotope distribution measurements as alpha particle detection in nuclear facilities or high energy physics experiments. For this purpose, we developed an ultrahigh resolution radiation imaging system using an optical fiber structure scintillator plate. We used a ~1-μm diameter fiber structured GdAlO_3_:Ce (GAP) /α-Al_2_O_3_ scintillator plate to reduce the light spread. The fiber structured scintillator plate was optically coupled to a tapered optical fiber plate to magnify the image and combined with a lens-based high sensitivity CCD camera. We observed the images of alpha particles with a spatial resolution of ~25 μm. For the beta particles, the images had various shapes, and the trajectories of the electrons were clearly observed in the images. For the gamma photons, the images also had various shapes, and the trajectories of the secondary electrons were observed in some of the images. These results show that combining an optical fiber structure scintillator plate with a tapered optical fiber plate and a high sensitivity CCD camera achieved ultrahigh resolution and is a promising method to observe the images of the interactions of radiation in a scintillator.

## Introduction

Recently there is increased demand for real-time, high resolution imaging for radiations. High resolution imaging of radiations is required for radioisotope distribution measurements, including for sliced subjects of animals or cells^1^, alpha particle detection in nuclear facilities^2,3^, or high energy physics experiments^4,5^. For alpha particle imaging detectors, an image intensifier combined with a scintillator plate was developed^6,7^. Other photodetectors, such as a position sensitive photomultiplier (PSPMT), a silicon photomultiplier (Si-PM) array, or a charge coupled device (CCD) camera, were also combined with scintillators to develop alpha particle imaging detectors^8–11^. For beta particle imaging detectors, systems made of an image intensifier combined with a scintillator plate were developed^7,12^ as well as a gas scintillator chamber or a scintillator plate combined with CCD cameras^13,14^. High resolution gamma photon imaging detectors commonly used small scintillator pixels optically coupled to a Si-PM array for ultrahigh resolution small animal PET systems^15–17^. A high resolution semiconductor-based imaging detector was also developed for a Compton camera^18^. Although using scintillator plates with photodetector arrays or high sensitivity CCD cameras is one method to obtain high resolution images of radiation, the scintillation light produced in the transparent scintillators cannot exit from the scintillator due to the total internal reflection inside the scintillator because of its high refractive index. Thus most of the scintillation light produced in the scintillator is not used for estimating the position of the interaction of the radiation.

**Figure 1:**
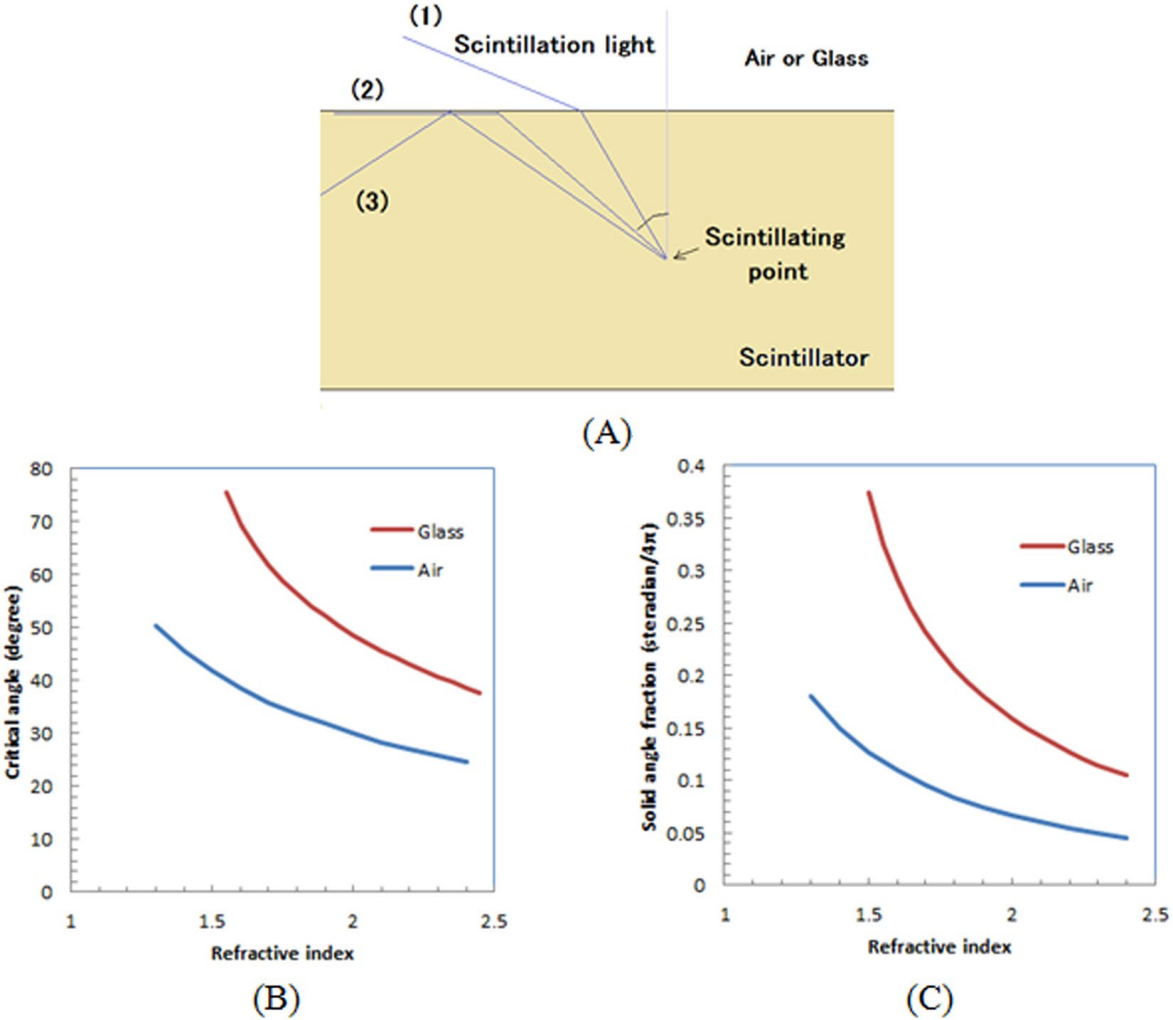
Schematic drawing of reflection of scintillation light inside scintillator plate (**A**), calculated critical angle (**B**), and solid angle fraction (**C**) as function of refraction index of scintillator.

Figure 1(A) shows a schematic drawing of the problem when a scintillator plate is used for the imaging of radiation. The scintillation light produced in the scintillator can leave the scintillator when the angle of the scintillation light to the vertical axis is small ((1) in Fig. 1(A)). However, the scintillation light moves parallel to the surface of the scintillator plate ((2) in Fig. 1(A)). This is called the critical angle. When it is larger than the critical angle, as shown (3) in Fig. 1(A), the scintillation light is reflected inside the scintillator by the total internal reflection and cannot escape.

We show the calculated critical angle as a function of the refractive index of the scintillator in Fig. 1(B) for air (refractive index: 1) and a typical example of glass (refractive index: 1.5). As the refractive index of the scintillator increases, the critical angle decreases. We also show the calculated solid angle fraction as a function of the refractive index of the scintillator for air and glass in Fig. 1(C). For example, for a scintillator with a refractive index of 1.9, only 7.5% of the scintillation photons can come out from the scintillator plate to air and 19.3% to glass. The rest of the scintillation light is reflected inside the scintillator and becomes stray light and degrades the spatial resolution or decreases the image’s contrast.

One possible method to reduce this effect is the use of a pixelated scintillator, which is cut with a dicing method^19–21^. However, the scintillator’s pixel size remains currently constrained to ~0.2 mm due to technical limitations. Another method is with a columnar scintillator made of CsI:Tl that is mainly used for an X-ray imaging system. However, even with a CsI:Tl columnar film, the spatial resolution degrades because increasing the thickness scatters more light in the scintillator.

To solve the problem, an optical fiber structured scintillator was developed^22–24^ from sub-micron diameter scintillator fibers made of a high refractive index surrounded by another scintillator material with a lower refractive index and formed into an optical fiber structure. By using the optical fiber structured scintillator, the scintillation light produced in the scintillator is emitted to the upper side of the scintillator plate and a fraction of the light used for imaging can be increased. We used an optical fiber structure scintillator plate (a fiber scintillator plate) combined with a CCD camera to obtain high resolution images of radiation. In addition, we used a tapered optical fiber plate between the fiber scintillator plate and the CCD camera to magnify the scintillation image in the former to improve the spatial resolution for a ultrahigh resolution radiation imaging system.

## Fiber scintillator plate

**Figure 2:**
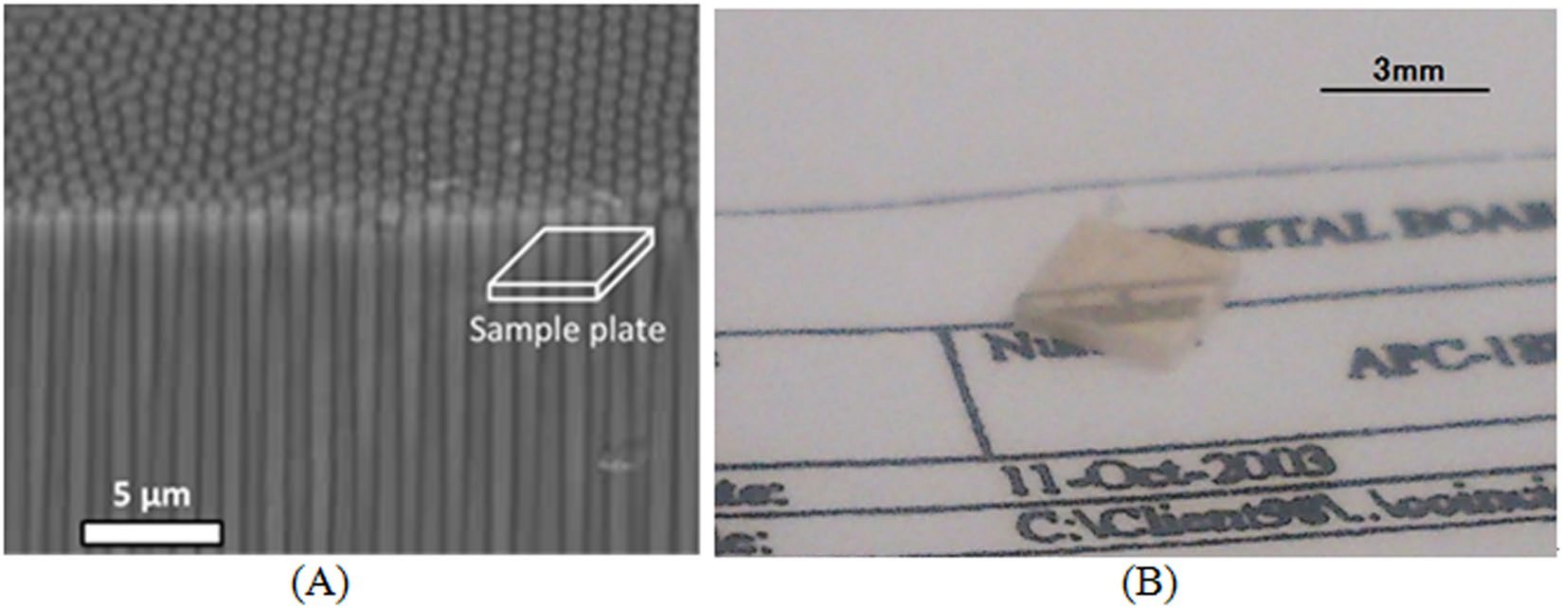
Schematic drawing of bird’s-eye view of SEM image of fiber scintillator plate (**A**). White fiber scintillators are GAP phase, and black matrices areα-Al_2_O_3_ phase in the SEM image. Photo of fiber scintillator plate used for imaging system on piece of paper (**B**).

We show a schematic cross-sectional view of a fiber scintillator plate (Fig. 2(A)) that was composed of Tb doped GdAlO_3_ (GAP: gadolinium aluminum perovskite) combined withα-Al_2_O_3_ for producing submicron-diameter phase-separated scintillator fibers. The refractive index of GAP is 2.05 and that ofα-Al_2_O_3_ is 1.79; thus an optical fiber structure can be formed when the GAP is used for the core andα-Al_2_O_3_ is for the clad. The average diameter of the scintillator fibers was ~1 μm.

Figure 2(B) shows a photo of a fiber scintillator plate made of GAP/α-Al_2_O_3_ used for our developed imaging system. The 3.0 mm × 2.5 mm × 0.5 mm thick plate was made by a micro-pulling down (α-PD) method at Tohoku University. We can observe the pop-up image of the characters and the lines on a piece of paper in the upper side of the scintillator plate in Fig. 2(B). We summarize the physical properties of the GAP/α-Al_2_O_3_ fiber scintillator plate of our imaging system in Table 1.

**Table 1:** Physical properties of GAP/α-Al_2_O_3_ fiber scintillator plate.

Light production	Emission light wavelength	Density	Decay time
~50,000 photons/MeV	450–700 nm	5.76 g/cm^3^	~1 ms

## Configurations of ultrahigh resolution radiation imaging system

**Figure 3:**
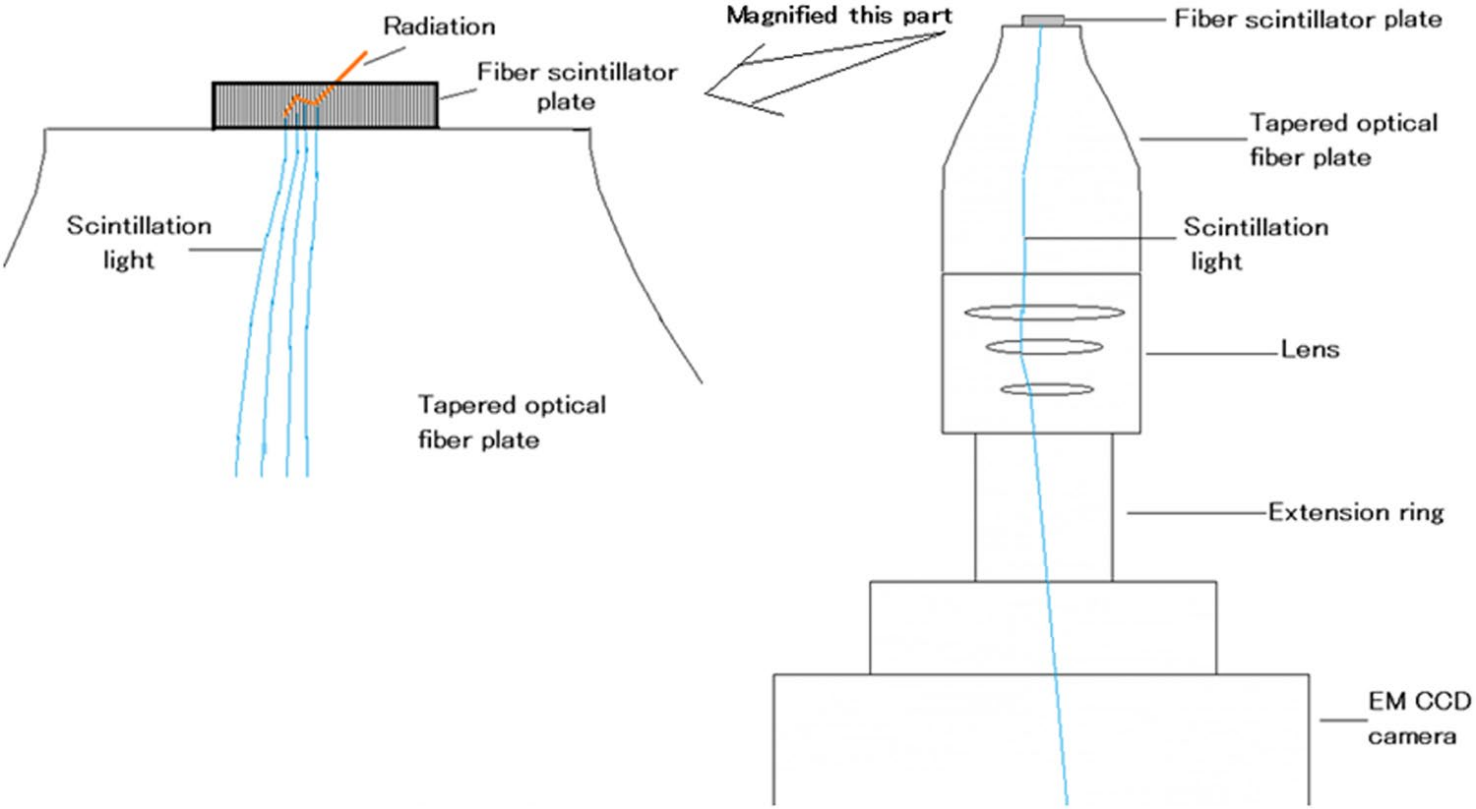
Schematic drawing of configuration of ultrahigh resolution radiation imaging system.

We show a schematic drawing of our ultrahigh resolution radiation imaging system in Fig. 3. It consists of a fiber scintillator plate, a tapered optical fiber plate, a lens, an extension ring, and an electron-multiplied (EM) charge coupled device (CCD) camera. The fiber scintillator plate is optically coupled to the tapered optical fiber plate. The tapered optical fiber plate, the lens, and the EM CCD camera are air-coupled (there are spaces between them).

When such radiation as alpha particles, beta particles, or gamma photons is detected by the fiber scintillator plate, the produced scintillator light travels toward the lower side of the scintillator plate. The scintillating position in the scintillator parallel to the plane is projected to the lower side of the scintillator plate because it has a fiber structure. The scintillation image is transferred to the tapered optical fiber plate and magnified while retaining the position information. The magnified scintillation image at the lower part of the tapered optical fiber plate is imaged by the lens-based EM-CCD camera. To reduce the distance from the tapered optical fiber plate and the lens, we placed an extension ring between the lens and the EM-CCD camera. With these configurations, the distribution of the scintillation in the fiber scintillator plate is magnified and detected by the EM-CCD camera.

## Developed ultrahigh resolution radiation imaging system

**Figure 4:**
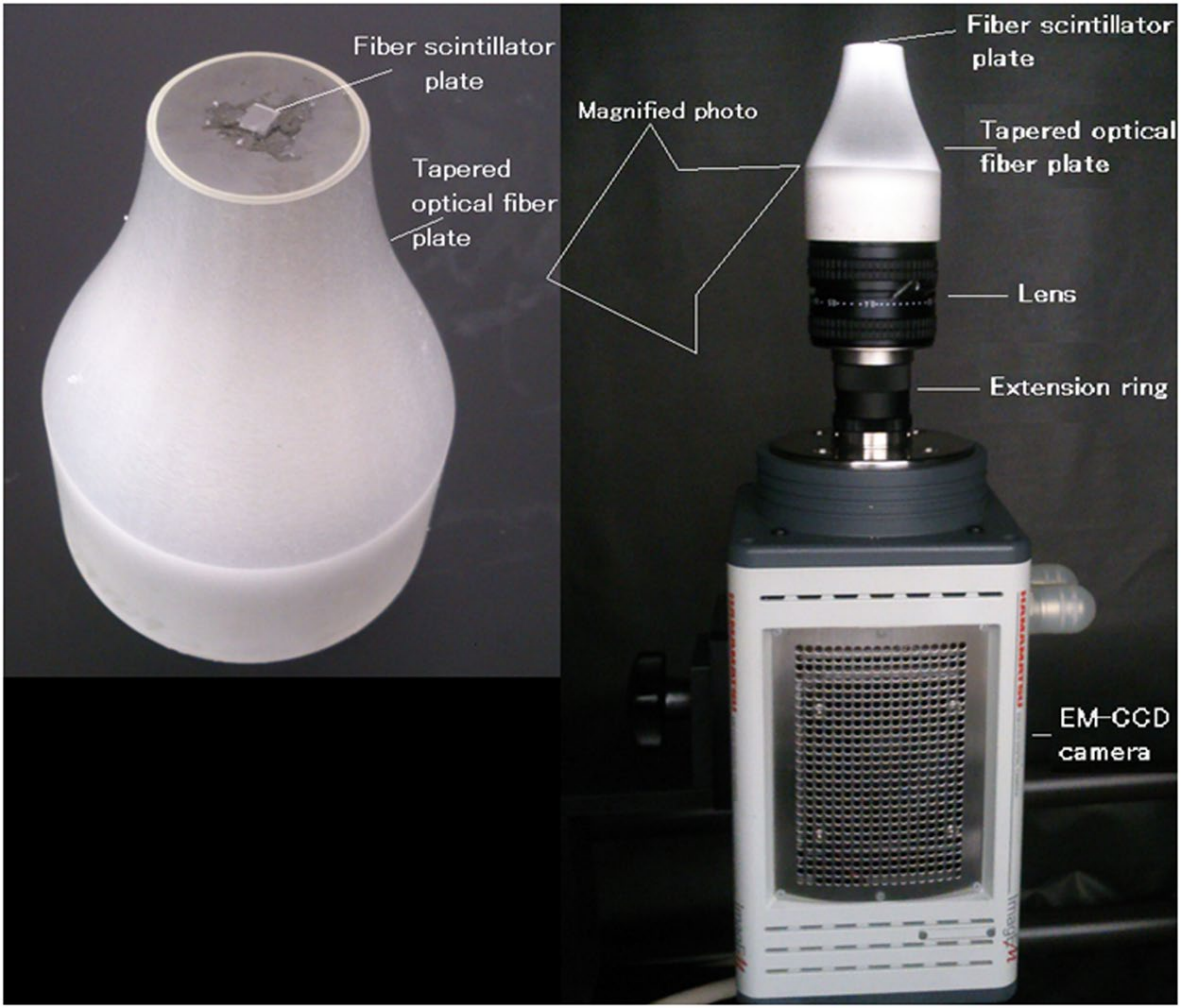
Developed ultrahigh resolution radiation imaging system; right side photo shows whole system and left side is its upper part.

The developed ultrahigh resolution radiation imaging system is shown in Fig. 4. The fiber scintillator plate was a 2.5 mm × 3.0 mm, 0.5-mm thick GAP/α-Al_2_O_3_ plate whose size was identical as that shown in Fig. 2(B). It was optically coupled to the center of the tapered optical fiber plate with a silicone rubber (KE-420, Sin-etsu Silicone, Japan) (Fig. 4 (left)). During the measurements, the scintillator plate and the upper side of the tapered optical fiber plate were covered by a thin aluminized Mylar film for a reflector when we measured the radiation. Because the fiber size of the scintillator plate (1 μm) was much smaller than the input size of the tapered fiber plate (~20 μm), no special care was required to align them.

The size of the upper side of the tapered optical fiber plate was 20 mm in diameter and the lower size was 50 mm in diameter (SCHOTT North America, USA). An optical fiber plate consists of a number of tightly packed optical fibers, and the image formed on one side is transferred to the other side. Since the fiber plate used for the system has a tapered shape, the image formed on the smaller side is magnified at the larger side. The diameters of the optical fibers of the tapered optical fiber plate were ~20 μm on the upper side, ~50 μm on the lower side, and the numerical aperture was 1.0. The tapered optical fiber plate was identical to that used for one of the beta cameras^25^. The image projected on the upper side was magnified 2.5 times at the surface of the lower side.

A F-0.95 C-mount type lens (Schneider, Xenon 0.95/25, Germany) was focused at the lower surface of the tapered optical fiber plate that was combined with the EM-CCD camera. A 25-mm long extension ring was used between the lens and the EM-CCD camera. With the extension ring, the distance between the tapered optical fiber plate and the lens was reduced.

The EM-CCD camera was an air-cooled type (Hamamatsu Photonics, ImagEM C9100-13, Hamamatsu, Japan) that operated at −65 °C. The following are the primary parameters used for the measurements:1 × 1 or 2 × 2 rebinning, sensitivity: 255, EM mode: off, readout speed: 3, and image size: 512 × 512 or 256 × 256. The whole system was set inside a black box surrounded by two layers of light tight curtains. The pixel size of the image was 5 μm for the 512 × 512 mode, 10 μm for the 256 × 256 mode, and the field-of-view (FOV) was 2.5 mm × 2.5 mm. The EM-CCD camera was identical as that used for the optical light detection of positron distribution^26^. We kept these parameters constant during imaging except for the acquisition time.

## Results

### Imaging experiments of radiation

1. Optical images of the transparent materials measured by the imaging system
  We show an optical image of an LGSO block in Fig. 5(A). In it, LGSO pixels (1.1 mm × 1.2 mm) and the reflector (~50 μm width) are clearly observed. An optical image of the GAGG pixels is shown in Fig. 5(B). The GAGG pixels (0.2 mm × 0.2 mm) are dark, and the slits (0.1 mm width) are bright. Both 0.1 to 0.2 mm structures are clearly visible in the images. We show an optical image of a colored printed plastic sheet in Fig. 5(C). The dots of the colored parts (80 μm diameter) and the spaces (~30 μm) are clearly resolved. We also show an image without transparent material to observe a defect in the fiber scintillator plate (Fig. 5(D)).

**Figure 5:**
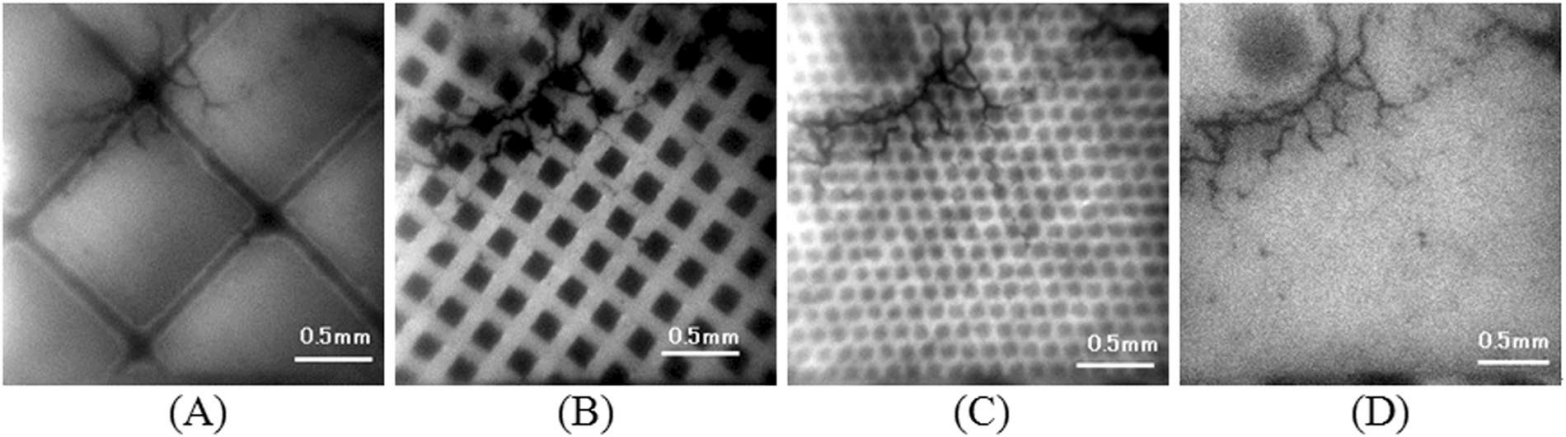
Optical images of transparent materials measured by imaging system with room light: LGSO scintillator block (**A**), GAGG pixel scintillator (**B**), and colored printed plastic sheet (**C**). Image without transparent material is also shown in (**D**). Black irregular lines mainly observed in left upper side of images are defects in fiber scintillator plate.
2. Imaging of alpha particles
  Figure 6 shows images of alpha particles measured by our developed radiation imaging system with a 256 × 256 mode. They were displayed with a maximum intensity level of 2000. The alpha particle images were round in all of the images. We show other frame images of the alpha particles in the supplemental information.

**Figure 6:**
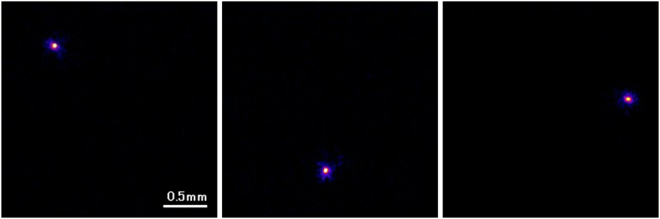
Images of typical alpha particles measured by developed imaging system.
  We show the average distribution profile of the alpha particle spots in Fig. 7 estimated for a 512 × 512 mode. The average spatial resolution for the 512 × 512 mode measured from the point spread functions of the alpha particle in the images was 25 ± 1 μm. The average spatial resolution for the 256 × 256 mode was 55 ± 6 μm FWHM.

**Figure 7:**
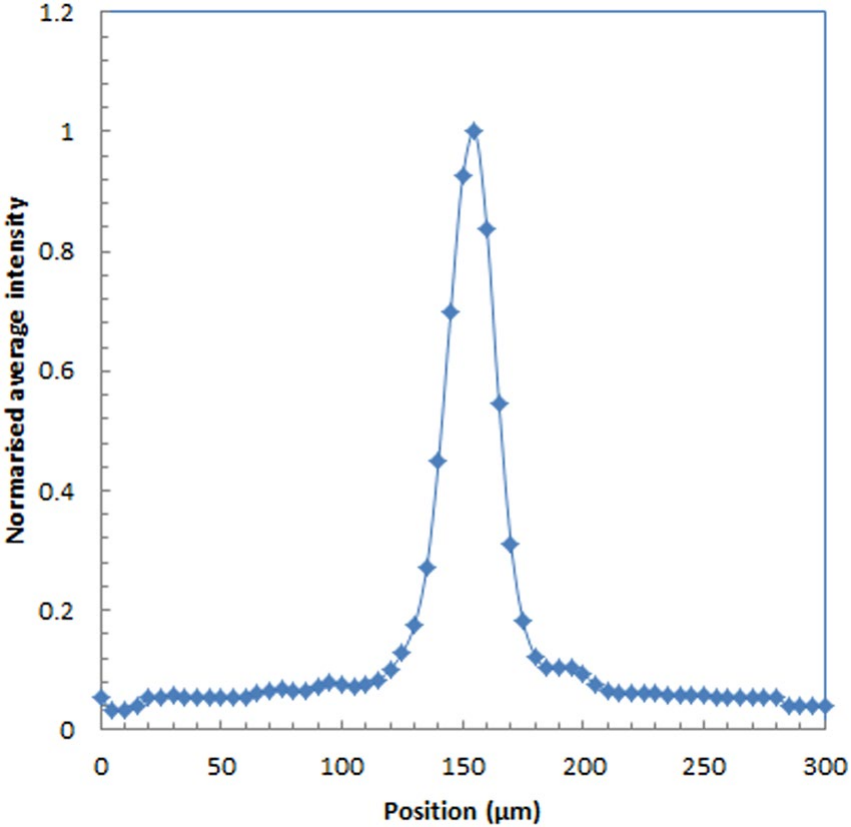
Intensity profile of alpha particle for 512 × 512 mode.
3. Imaging of beta particles
  Figure 8 shows three typical images of beta particles measured by the developed imaging system. The images were displayed with a maximum intensity level of 400. The images of the beta particles had many types of shapes, showing their trajectories. We also show other frame images of beta particles in the supplemental information.

**Figure 8:**
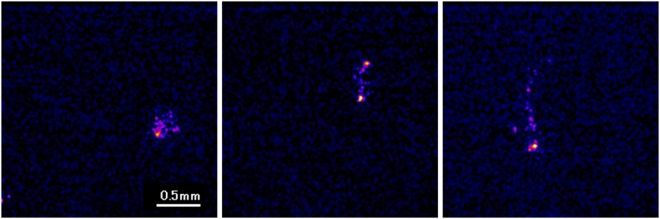
Images of typical beta particles measured by developed radiation imaging system.
4. Imaging of gamma photons
  Figure 9 shows images of typical gamma photons measured by the developed imaging system. The images were displayed with a maximum intensity level of 400. Most of the gamma photon images were round spots with some irregular distributions around the spots that show the secondary electron trajectories. We also show other frame images of gamma photons in the supplemental information.

**Figure 9:**
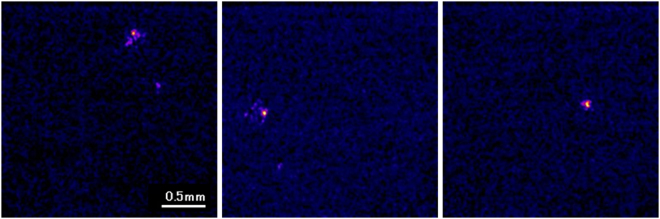
Images of typical gamma photons measured by developed radiation imaging system.
5. Intensity comparison of radiation
  The average maximum intensity for the alpha particles, the beta particles, and the gamma photons is shown in Fig. 10(A). The average maximum intensity of the spots in the images for the alpha particles was more than six times larger than those for the beta particles and the gamma photons. The evaluated maximum intensity distribution for the alpha particles, the beta particles, and the gamma photons is shown in Fig. 10(B). The maximum intensity for the alpha particles was distributed higher than those for the beta particles and the gamma photons.

**Figure 10:**
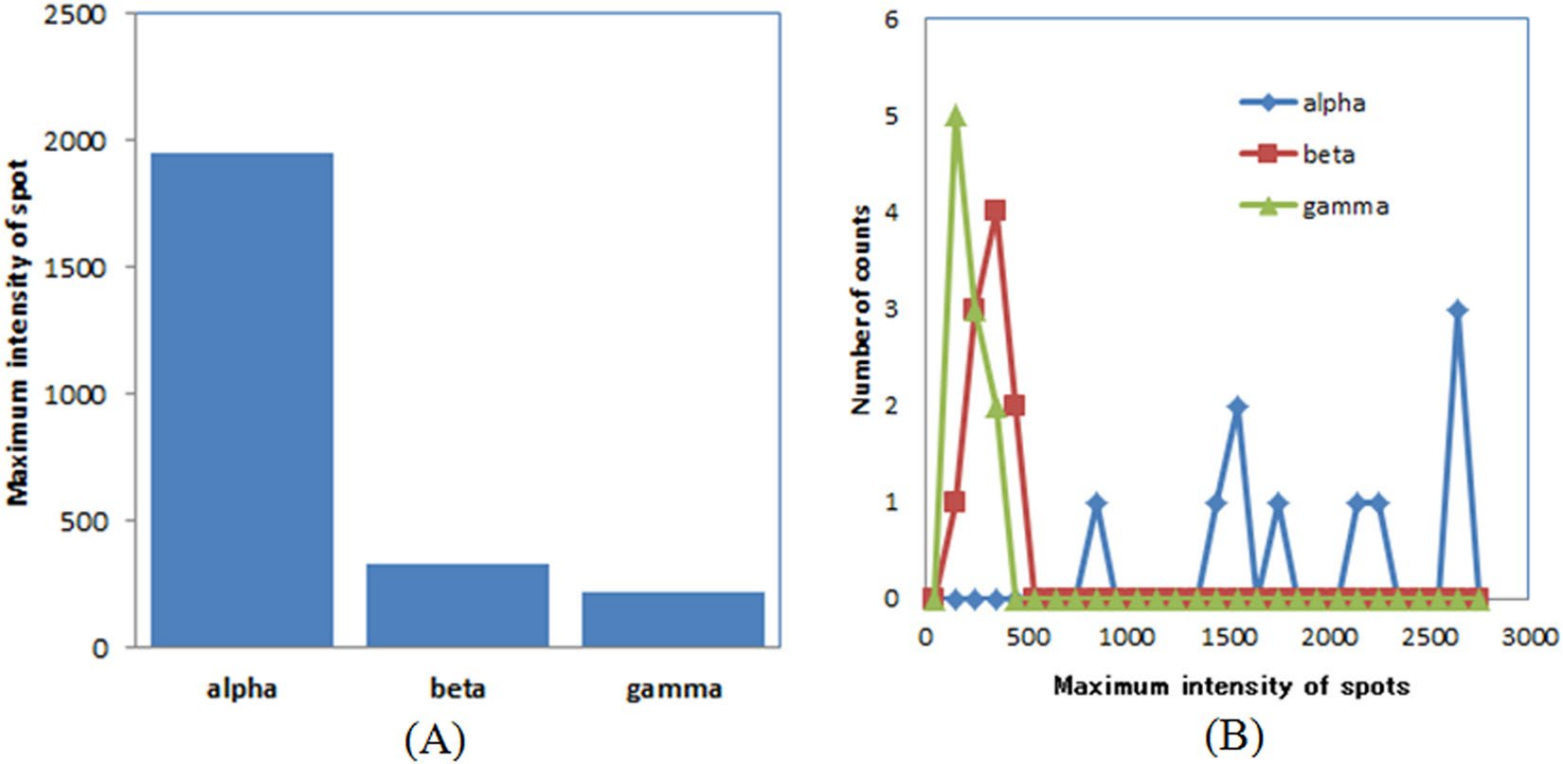
Average maximum intensity (**A**) and maximum intensity distribution (**B**) for alpha particles, beta particles, and gamma photons.

## Discussion

We successfully developed an ultrahigh resolution radiation imaging system using a fiber scintillator plate combined with a tapered optical fiber plate and an EM-CCD camera. With our system, we used a fiber scintillator plate that can increase the light output from the scintillator plate. We achieved ~25-μm FWHM spatial resolution without a microscope. Obtaining high resolution without a microscope is an advantage because it can reduce the size and the complexity of the imaging system.

Since the tapered optical fiber plate in our imaging system had an input and output size ratio of 2:5, the magnification ratio was 2.5. To increase the spatial resolution, using a tapered optical fiber plate with a larger magnification ratio is possible. For example, if we combine a tapered optical fiber plate with a magnification ratio of 5, a spatial resolution of ~10 μm may be realized.

Compared with common radiation imaging detectors, our system has higher resolution. Although its spatial resolution was not as high as the emulsion or the plastic-based track detectors that need chemical post processing to obtain distribution^29,30^, our system had the highest spatial resolution among real-time radiation imaging systems^6–18^. For example, an imaging system that combined ZnS(Ag) scintillator films with microchannel plate (MCP) image intensifiers achieved ~100 μm to 300 μm^6,7^ for alpha particles.

The optical images shown in Fig. 5 had high resolution, and fine structures of the transparent materials are clearly observed. These images indicate that the use of a fiber scintillator plate maintained the spatial resolution of the system. If we use a standard (non-fiber) plate type scintillator on the tapered optical fiber plate, the spatial resolution will be significantly degraded even with a thin scintillator plate. On the other hand, even with a fiber scintillator plate, the subject should be optically coupled to the fiber scintillator plate without space to obtain high resolution images of radiation in the subject.

The images of beta particles shown in Fig. 8 and some of the gamma photons shown in Fig. 9 are attractive because the trajectories of the electrons in the fiber scintillator plate can be observed in the images. The trajectories of the electrons in the images were not continuous but had some high intensity spots in the trajectories. Perhaps the electron’s energy was mainly absorbed at these high intensity points. So far, since it is difficult to image the electron trajectory on an event-by-event basis with scintillation imaging detectors, the developed system will be a new and useful tool in radiation detection and measurements.

The intensity of the spots in the images of the alpha particles is much higher than those for the beta particles and the gamma photons shown in Fig. 10(A) and (B). This is because the energy of the alpha particles was higher (5.5 MeV) than that of the beta particles (maximum energy: 546 keV and 2.28 MeV) or the gamma photons (mainly 511 keV) as well as because the spatial distribution of the intensity had a round small spot shape for the alpha particles. In fact, the range of the alpha particles for the experiments was around 10 μm. Based on our results, alpha particles can be confidently separated from beta particles and gamma photons. On the other hand, the separation of beta particles and gamma photons is difficult due to the maximum intensity of the spots because the intensity levels of these two types of radiation were similar. Since the distribution of the beta particles from Sr-Y-90 was wider than the gamma photons from Na-22, probably because of the higher energy for the beta particles from Sr-Y-90, it may be possible to separate these two by the shape of the spots in the images.

Commonly, since a CCD sensor is sensitive to radiation and high intensity spots due to the direct detection of radiation sources, environmental radiation and cosmic rays are sometimes observed with a long exposure time. However, with a shorter exposure time (less than 1 s) and with the low activity radiation sources in our measurements, these noise spots were rarely observed. Damage to the CCD camera will not be a problem in our experiments due to a weak radioactivity source of MBq order. However, with the exposure of the radiation therapy level, serious damage will be accumulated in the CCD sensor as well as in electronics and personal computers (PC). No detection of cosmic rays has been observed with a shorter acquisition time than 1 s.

The spatial resolution of the developed imaging system for alpha particles is probably determined by the fiber size of the input part of the tapered fiber plate (20 μm). Since the estimated range of the 5.5 MeV alpha particles in the scintillator was ~10 μm, we may be able to improve the spatial resolution of the imaging system with a tapered fiber plate with a smaller fiber size and a higher magnification ratio.

Possible applications of our developed radiation imaging system include high resolution imaging of radiation for alpha and beta particles and particle distribution measurements in living cells. Because the spatial resolution of our imaging system for alpha particles is potentially excellent and the upper side of the fiber scintillator plate can be optically transparent, it will be useful for imaging the alpha particles emitted from living cells with optical photos of the cells that accumulate the alpha particle emitters if the spatial resolution can be improved in the future. The imaging system will be a powerful tool for the research field of the alpha targeting radionuclide therapy^31–33^.

## Methods

### Image processing

We processed the acquired images using public domain software (ImageJ). For radiation images, noise spots due to the direct absorption of gamma photons, X-rays from the source or environmental background by the CCD image sensor were eliminated using high-intensity and small-pixel information. For each measured image, we subtracted the offset value of the CCD image.

### Imaging experiments of radiation

First, we checked the optical imaging performance using transparent materials set on the detector surface without aluminized Mylar with room light on. Then we imaged alpha particles, beta particles and gamma photons for the imaging experiments of the developed radiation imaging system.

1. Optical images of the transparent materials measured by the imaging system
  To check the imaging system is properly worked, we conducted phantom imaging without radiation sources with room light on. Without aluminized Mylar on the fiber scintillator plate, we set transparent materials that had fine structures on the fiber scintillator plate and imaging was conducted with the imaging system. Because the room light intensity was too high to measure the images, we set the sensitivity gain to 1 for the imaging.
  We show the transparent materials used for the imaging test in Fig. 11. The transparent materials used were LGSO scintillator block which had size of 1.1 mm × 1.2 mm pixels with ~50 μm reflector between pixels (left side in Fig. 11) which was previously used for a Si-PM based PET system^27^, GAGG pixel scintillator made of dicing technique which had 0.2 mm pixels with 0.1 mm slits between pixels (middle in Fig. 11)^19–21,28^, and a color printed plastic sheet which had ~50 μm dots with 30 μm spaces between dots (right side in Fig. 11). One of these materials was set on the fiber scintillator plate and imaging was conducted to check the structures of these materials were observed. Because the scintillator plate had the fiber structure, the material structure on the upper side of the scintillator plate can be imaged by the CCD camera. Only a part of these materials were imaged with the system because the FOV of the imaging system was small. Images were acquired with the 256 × 256 mode.

**Figure 11:**
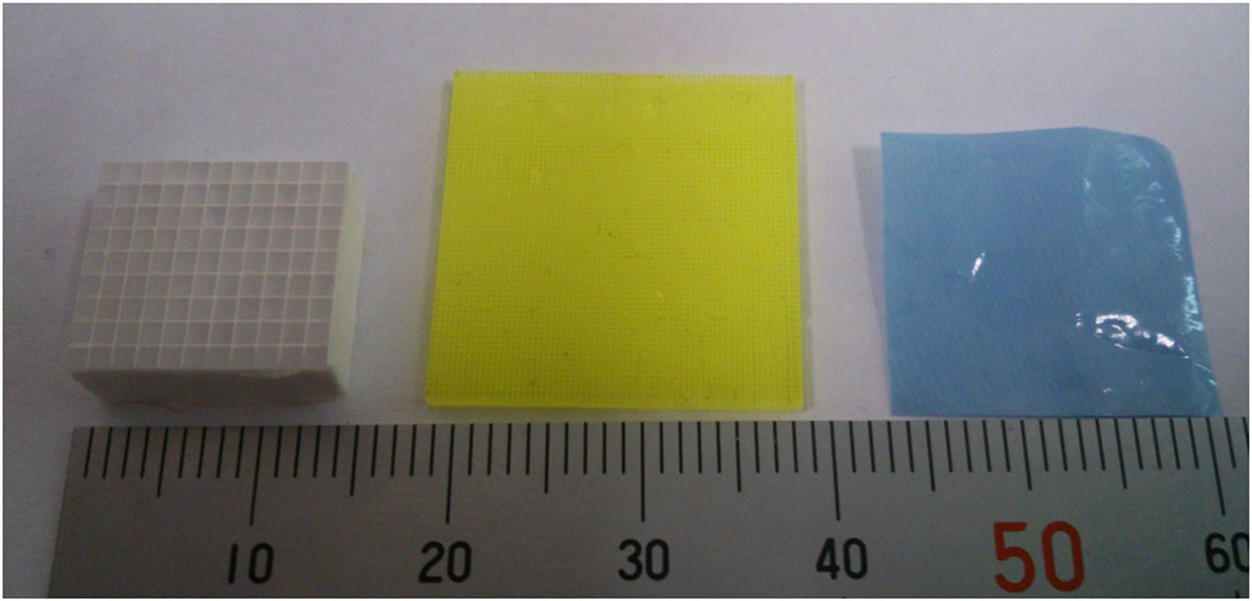
Transparent materials used for the imaging test of system: LGSO scintillator block which has size of 1.1 mm × 1.2 mm pixels with ~50 μm reflector between pixels (left), GAGG pixel scintillator which has 0.2 mm pixels with 0.1 mm slits between pixels (middle) and color printed plastic sheet which has 50 μm diameter dots with 30 μm spaces between dots (right).
2. Imaging of alpha particles
  For the imaging of alpha particles with the developed imaging system, we used Am-241 alpha source (5.5 MeV alpha particles). A 2k Bq of Am-241 alpha source was set above the fiber scintillator plate and imaging was conducted for 50 ms. Images were acquired both with 256 × 256 and 512 × 512 modes. We measured more than 10 images which has a spot of alpha particles in the image. After image processing, point spread function was measured to estimate the spatial resolution of the imaging system.
3. Imaging of beta particles
  For the imaging of beta particles, we used a Sr-Y-90 beta source (maximum energy: 546 keV and 2.28 MeV beta particles). A 100 Bq of Sr-Y-90 beta source was set above the fiber scintillator plate and imaging was conducted for 50 ms. Images were acquired with 256 × 256 mode. We measured the images with low count rate to measure only one beta particle in an image being detected. This was to confirm the shape of a beta particle trajectory. We measured more than 10 images which had a trajectory of beta particles in the image.
4. Imaging of gamma photons
  For the imaging of gamma photons, we used Na-22 source contained in aluminum container (mainly 511 keV gamma photons). A 30 k Bq of Na-22 gamma source was set above the fiber scintillator plate and imaging was conducted for 50 ms. Images were acquired with 256 × 256 mode. We measured the images with low count rate to measure only one gamma photon in an image was detected to confirm the shape of the secondary electron trajectory produced by the gamma photon. We measured more than 10 images which has a trajectory or a spot of gamma photon in the image.
5. Intensity comparison of radiations
  We evaluated the intensity of the spots in the images for alpha particles, beta particles and gamma photons to compare the intensity of these radiations. This was to check the possibility to distinguish the types of radiation from the intensity. We analyzed the maximum pixel counts of the spots in the images for alpha particles, beta particles and gamma photons and average intensity, and the intensity distribution were plotted.

## Electronic supplementary material

Supplemental information
