# Supplementary material for: Ultrahigh resolution radiation imaging system using an optical fiber structure scintillator plate
Source: Sci Rep. 2018 Feb 16;8:3194. doi: 10.1038/s41598-018-21500-z (PMC5816672; doi:10.1038/s41598-018-21500-z)
Supplement: Supplementary file 1 — Supplemental information [file 41598_2018_21500_MOESM1_ESM.doc]

**Supplemental information:**

**Ultrahigh resolution radiation imaging system using an optical fiber structure scintillator plate**

**Seiichi Yamamoto, Kei Kamada, and Akira Yoshikawa**

**Images of alpha, beta particles and gamma photons**


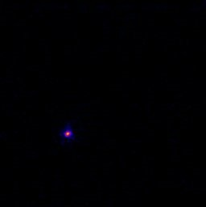

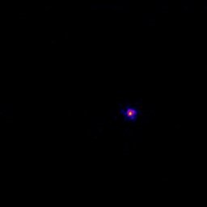

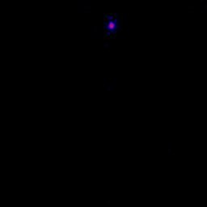

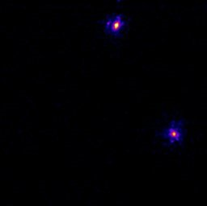

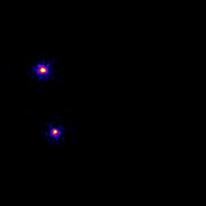


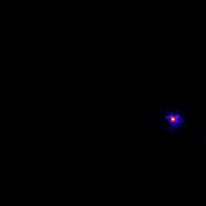

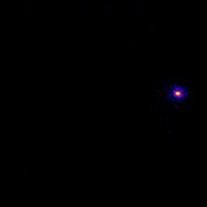

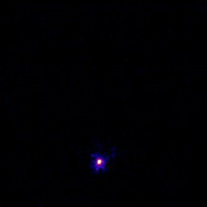

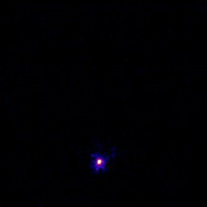

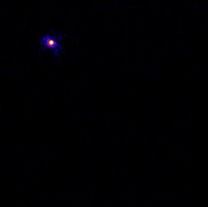


Figure S1 Set of images for Am-241 alpha particles measured by developed imaging system


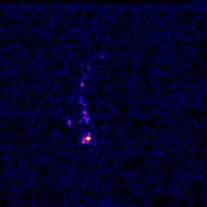

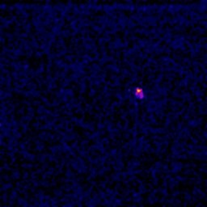

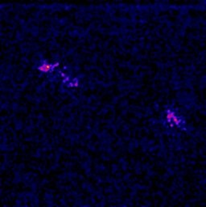

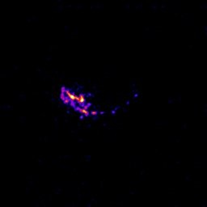

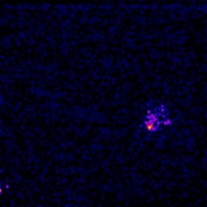


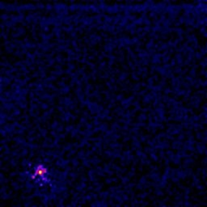

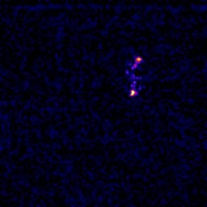

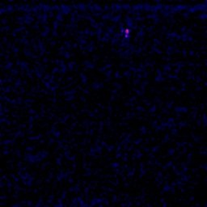

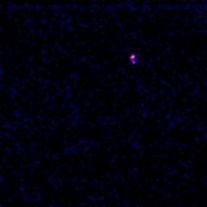

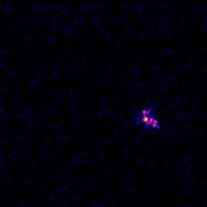


Figure S2 Set of images for Sr-Y-90 beta particles measured by developed imaging system


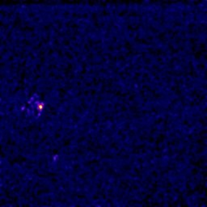

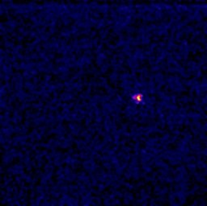

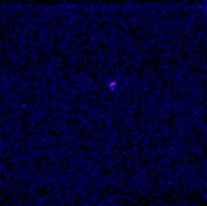

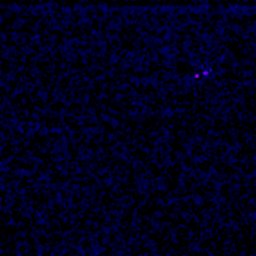

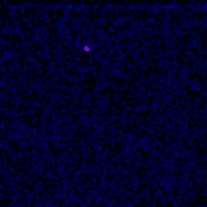


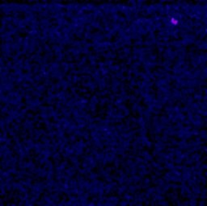

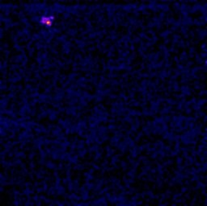

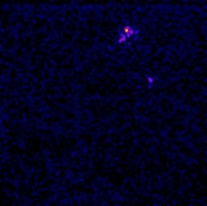

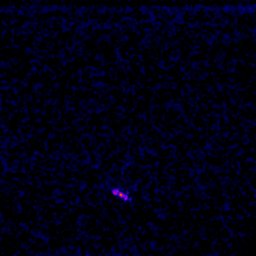

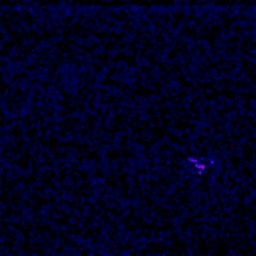


Figure S3 Set of images for Na-22 gamma photons measured by developed imaging system
